# Creosote in Vomiting

**Published:** 1847-12

**Authors:** 


### *Creosote in Vomiting.*

To the Editor of the Boston Medical and Surgical Journal.

Sir,—In the last No. of your Journal I found a few remarks, taken from the Buffalo Medical and Surgical Journal, in reference to the use of creosote in vomiting; and as the following case is one in point, I transmit it to you for pnblication.

A daughter of Dr. D. E. Brown, æt. 4 years, was taken on the 7th day of this month (October) with a slight chill, accompanied with obstinate vomiting, and followed by high fever, without any abatement of the sickness of the stomach. Calomel, morphine and camphor, severally and combined, together with the use of enemas, and of hot applications externally, were resorted to, and every means which the skill and experience of her father could devise were brought into requisition to allay the irritation of the stomach —but without success. For five days it continued to baffle every effort for its subjugation, the stomach rejecting every thing that was taken into it, even to a teaspoonful of water. On the fourth day of the disease, a slight evacuation from the bowels had been procured; the discharge was white and watery. On the evening of the 12th (the fifth day of the disease) I saw her for the first time. Her appearance was alarming in the extreme. She was lying in a semi-comatose state, her eyes fixed and glassy, her countenance pale and Hippocratic, respiration slow and laborious, pulse frequent, and so small and thread-like as to be scarcely perceptible, the extremities and the whole surface of the body cold. Every thing taken upon the stomach would be rejected in a short time, and spontaneous paroxysms of vomiting were recurring every few minutes. The use of the creosote was now agreed upon. One drop in a little syrup was given, but was thrown up in about three minutes. Two drops more were immediately administered, which were retained. The lower bowels were now distended with enemas of milk and water, in which were contained a little common salt, and frictions and hot applications externally were made use of. From this time, the stomach became and continued quiet, and in about an hour the injections came away, bringing with them a considerable quantity of fecal matter. Two grains of quinine and twenty drops of brandy in a tablespoonful of water were now given. The pulse gradually lessened in frequency, and increased in strength and volume; the eyes and countenance assumed their natural appearance, the breathing became easier, and the surface and extremities warm. Nothing further was done until morning, when a little chicken broth was given, and an enema, containing five grains of quinine, thrown up the bowels. The broth was retained upon the stomach, and constituted the first article of nourishment that had found a moment's resting place there for five days. From this time her recovery was rapid and complete, not the slightest return of the vomiting having taken place since the second dose of creosote was given.

I have had occasion to make use of the creosote in several cases of obstinate vomiting, prior to the one now reported, but never before have I witnessed so happy an exhibition of its virtues.—Where a high degree of arterial excitement, with a flushed countenance and hot skin, accompany the morbid action of the stomach, I should be very much inclined to doubt the propriety of its administration, but when the system is prostrated, the pulse thread-like, the countenance bloodless and the extremities cold, it may be resorted to with a prospect of success which but few if any other agents can afford.

Schoolcraft, Mich., Oct. 16th, 1847. A. W. Mack, M. D.

